# Synthesis and DC Electrical Conductivity of Nanocomposites Based on Poly(1-vinyl-1,2,4-triazole) and Thermoelectric Tellurium Nanoparticles

**DOI:** 10.3390/ma16134676

**Published:** 2023-06-28

**Authors:** Anna V. Zhmurova, Galina F. Prozorova, Svetlana A. Korzhova, Alexander S. Pozdnyakov, Marina V. Zvereva

**Affiliations:** A. E. Favorsky Irkutsk Institute of Chemistry, Siberian Branch of Russian Academy of Sciences, Favorsky 1, 664033 Irkutsk, Russia

**Keywords:** polymer nanocomposite, dielectric polymer, poly(1-vinyl-1,2,4-triazole), thermoelectric tellurium nanoparticles, DC electrical conductivity

## Abstract

In this work, the structural characteristics and DC electrical conductivity of firstly synthesized organic–inorganic nanocomposites of thermoelectric Te^0^ nanoparticles (1.4, 2.8, 4.3 wt%) and poly(1-vinyl-1,2,4-triazole) (PVT) were analyzed. The composites were characterized by high-resolution transmission electron microscopy, X-ray diffractometry, UV-Vis spectroscopy, and dynamic light scattering analysis. The study results showed that the nanocomposite nanoparticles distributed in the polymer matrix had a shape close to spherical and an average size of 4–18 nm. The average size of the nanoparticles was determined using the Brus model relation. The optical band gap applied in the model was determined on the basis of UV-Vis data by the Tauc method and the 10% absorption method. The values obtained varied between 2.9 and 5.1 nm. These values are in good agreement with the values of the nanoparticle size, which are typical for their fractions presented in the nanocomposite. The characteristic sizes of the nanoparticles in the fractions obtained from the Pesika size distribution data were 4.6, 4.9, and 5.0 nm for the nanocomposites with percentages of 1.4, 2.8, and 4.3%, respectively. The DC electrical conductivity of the nanocomposites was measured by a two-probe method in the temperature range of 25–80 °C. It was found that the formation of an inorganic nanophase in the PVT polymer as well as an increase in the average size of nanoparticles led to an increase in the DC conductivity over the entire temperature range. The results revealed that the DC electrical conductivity of nanocomposites with a Tellurium content of 2.8, 4.3 wt% at 80 °C becomes higher than the conventional boundary of 10^−10^ S/cm separating dielectrics and semiconductors.

## 1. Introduction

Electronic devices that consume electrical energy and inevitably produce parasitic heat are now widespread. The demand for systems that convert heat into electrical energy is therefore growing critically. It is known that the construction of the systems is possible in the form of a combination of thermoelectric coolers (TEC) and thermoelectric generators (TEG) [1]. The conversion efficiency of TEC and TEG is directly related to the thermoelectric figure of merit (ZT) of the materials implemented as thermoelectric cell legs. Currently, an active search for high-ZT thermoelectrics is underway. Particular attention is paid to the development of polymer-based thermoelectrics (PTs) due to their advantages over completely inorganic systems [2], such as low cost, environmental friendliness and diversity of synthesis methods, low thermal conductivity, relatively light weight, mechanical flexibility, etc. [3]. One important class of PTs are organo–inorganic nanocomposites that combine the thermoelectric properties of their inorganic nanophase (metal chalcogenides and oxides as well as elemental chalcogenes, in particular Te^0^ [4,5,6,7,8,9,10]) with the performance and mechanical properties of polymer matrices. The PTs exhibit a relatively high Seebeck coefficient and low thermal conductivity. The use of insulating polymers as matrices for nanocomposites compared to conducting polymers allows the production of cheaper and more stable thermoelectrics with features important in practice (flexibility, extensibility, thermoplasticity).

The most widely applied polymers in PT creation are polyvinylidene fluoride [11], polyvinyl alcohol [12], polymethyl methacrylate [6], cellulose [5], and polyvinylpyrrolidone [13], etc. According to the literature, the direct incorporation of nanoparticles into the polymer matrix is achieved by combining the pre-synthesized nanoparticles and polymer in a solution with a subsequent drop casting, hot compaction [11,14], or vacuum filtration [11], mechanical pressing [11], or screen printing [15]. It should be noted that the above methods of PT creation are time consuming, often based on the application of special equipment providing sonication [16,17], high-pressure action [13], and high temperatures [10,12], as well as the use of organic toxic solvents and an extensive range of nanoparticle precursors (NaBr [5], Na_2_TeO_3_ [10], NaBH_4_ [10], Na_2_SeO_3_ [11], TeO_2_ [6,14], SeO_2_ [13], NH_2_OH [14], CuSO_4_ [18]), which have toxic and environmentally unfriendly properties.

In this regard, the use of previously developed simple and environmentally friendly methods of producing chalcogen containing nanoparticles [19,20], particularly Te^0^ ones from the commercially available bulk Te powder, as well as the application of the original polymer poly(1-vinyl-1,2,4-triazole) (PVT) in the form of a polymeric stabilizing matrix for the nanoparticle formation, seems highly promising for the synthesis of thermoelectric nanocomposites. The PVT exhibits a complex of practically important properties such as high hydrophilicity, solubility in polar organic solvents, ability for complexation and quaternization, chemical stability, biocompatibility, and thermal stability. In addition, triazole-containing polymers were previously experimentally found to behave as effective stabilizing matrices when forming metal-containing nanocomposites, exhibiting a synergy of unique polymer properties (solubility, biocompatibility, high coordination ability) and the optical, catalytic, and biological properties of metal nanoparticles [21,22,23,24]. At the same time, Te as a thermoelectric direct-gap semiconductor has been successfully used in the creation of polymeric organic–inorganic thermoelectrics with a p-type conductivity and both a conductive [25] and insulating matrix [10,26,27,28].

The paper presents the results of the polymer thermoelectrics synthesis of a Te nanophase and a PVT original polymer matrix with p-type conductivity and the study of the influence of nanocomposite structural features determined by the PVT: Te relation on their DC electrical conductivity.

## 2. Materials and Methods

### 2.1. Materials

NaOH (Reahim, Moscow, Russia), hydrazine hydrate (Reahim, Moscow, Russia), acetone (Reahim, Moscow, Russia), azobisisobutyronitrile (Sigma Aldrich, Burlington, VT, USA), dimethylformamide (Reahim, Moscow, Russia), and tellurium powder (Thermo Fisher, Kandel, Germany) were used without additional purification.

### 2.2. Methods

#### 2.2.1. Synthesis of PVT

The synthesis of PVT was carried out by a radical polymerization method of 1-vinyl-1,2,4-triazole (VT) in the presence of an azobisisobutyronitrile (AIBN) initiator in a dimethylformamide (DMFA) medium under an argon atmosphere and a temperature of 60 °C during 6 h, according to the protocol detailed in the paper [24].

**Figure 1:**
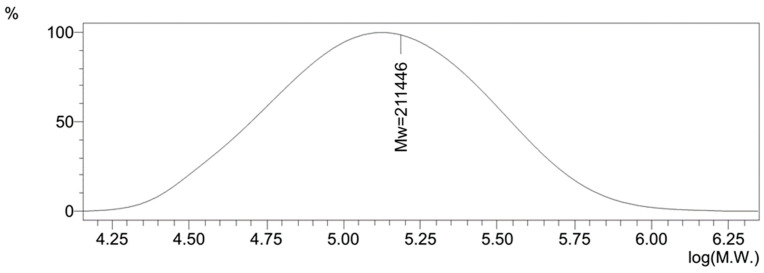
The molecular weight distribution of PVT.

Briefly, VT (1.5 g, 16.0 mmol), AIBN (0.015 g, 0.09 mmol), and DMFA (1.0 g) were placed in a glass ampoule, flasked with argon, then sealed and incubated in the thermostat at 60 °C for 6 h. The resulting PVT was isolated and purified by a double resuspension in a mixture of ethanol and acetone (1:2), and then dried to a constant mass in a vacuum oven over phosphorus pentoxide at 50 °C. The average molecular weight of the polymer was 110 kDa and the weight average molecular weight was 211 kDa. The polydispersity coefficient was 1.92 (Figure 1). The PVT obtained in a 92% yield was a white powder, well soluble in water, DMF, and DMSO. The calculated % were C 50.52; H 5.26; and N 44.22 The found % were C 50.61; H 5.25; and N 44.14.

**Figure 2:**
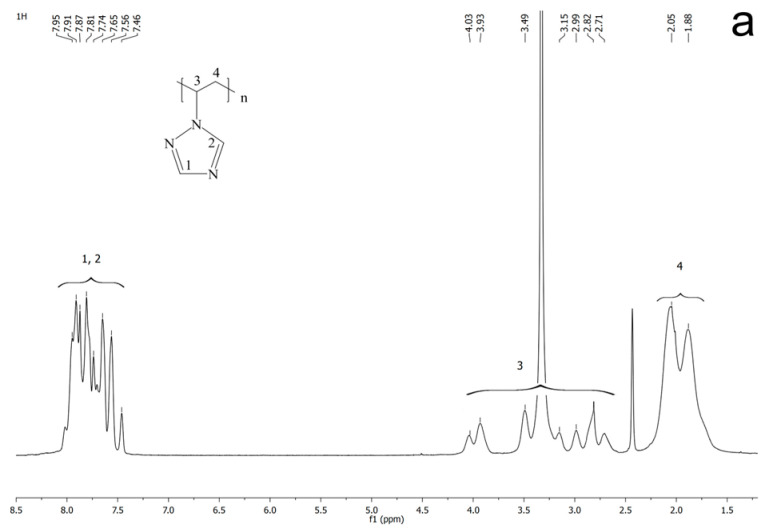

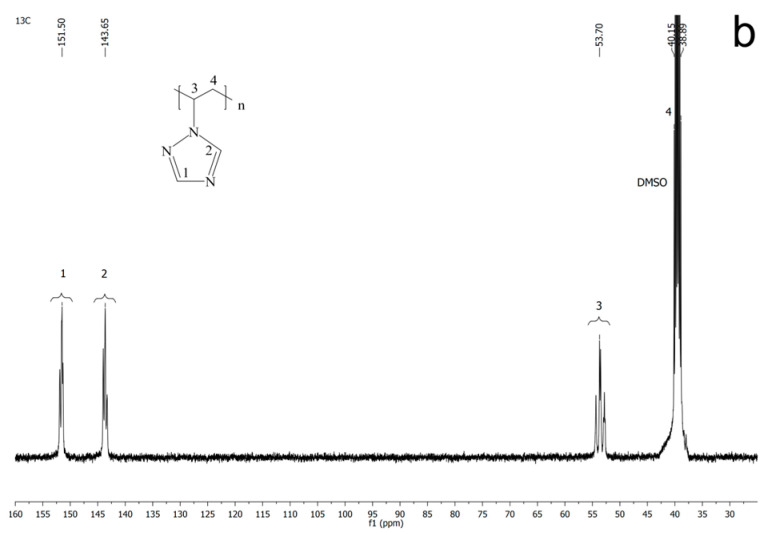
The ^1^H (**a**) and ^13^C NMR (**b**) spectra of PVT.

Using the method of nuclear magnetic resonance ^1^H and ^13^C, the structure of the obtained polymer was confirmed (Figure 2). The ^1^H NMR (DMSO- *d_6_*, δ, ppm) was 8.08–7.41 (br m., 2H, triazole ring), 4.15–2.66 (br m., 1H, CH in the polymer backbone), 2.25–1.60 (br, 2H, CH_2_ in the polymer backbone); the ^13^C NMR (DMSO- *d_6_*, δ, ppm) was 152.50–150.50, 145.00–142.5 (CH, triazole ring), 55.00–52.20 (CH in the polymer backbone), and 41.40–38.20 (probably the signals of carbon atoms of methylene groups CH_2_ of the polymer backbone overlap with solvent signals).

#### 2.2.2. Synthesis of PVT–Te^0^NPs

To synthesize the PVT–Te^0^NPs, the PVT (1 g) and distilled water (50 mL) were placed in the 100 mL flask. The reaction mixture was then stirred for 3 h at the temperature of 40 °C until the PVT was completely dissolved. The solution containing PVT was cooled to room temperature. The reaction mixture with tellurium anions (120–400 µL) that were pre-synthesized in accordance with the technique described in the articles [19,20] was added into a Te solution flask under constant stirring with a magnetic stirrer. The formation of the Te^0^ nanoparticles was identified by a change in the color of the reaction mixture from colorless (native PVT solution) or red–purple (reaction mixture containing Te^2−^ anions) to gray. The synthesis duration was 25 min, after which the reaction mixture was precipitated in acetone cooled to 7 °C. The resulting fine precipitate was separated by centrifugation at 4000 rpm at 9 °C, washed three times with acetone, and dried in a vacuum at room temperature. For the PVT–Te^0^NP (1.4%), the yield was 62%; calculated % were C 49.53; H 5.50; N 43.27; and Te 1.7. The found % were C 49.64; H 5.52; N 43.44; and Te 1.4. For the PVT–Te^0^NPs (2.8%), the yield was 71%; the calculated % were C 48.05; H 5.36; N 43.19; and Te 3.4. The found % were C 48.95; H 5.56; N 42.71; Te 2.80. For the PVT–Te^0^NPs (4.3%), the yield was 58%; the calculated % were C 48.00; H 5.56; N 41.04; and Te 5.4. The found % were C 48.30; H 5.67; N 41.73; and Te 4.3.

### 2.3. Equipment

#### 2.3.1. Elemental Analysis (EA)

The elemental composition was determined by X-ray energy dispersive microanalysis using a Hitachi TM 3000 scanning electron microscope (Tokyo, Japan) with an SDD XFlash 430-4 X-ray detector and a Thermo Fisher Scientific Flash 2000 CHNS analyzer (Kandel, Germany).

#### 2.3.2. X-ray Diffraction (XRD) Analysis

The XRD study was performed on a Bruker D8 ADVANCE diffractometer (Billerica, MA, USA) equipped with a Hebbel mirror, with Cu radiation in the locked coupled mode, with an exposure of 1 s for phase analysis and 3 s for the estimation of the cell parameter and the coherent scattering region size.

#### 2.3.3. UV-Vis Spectroscopy

The UV-Vis spectra of 0.025% water solutions of PVT and the nanocomposites were recorded relative to distilled water in a 1 cm quartz cell on a Perkin Elmer LAMBDA 35 UV-Vis spectrophotometer (Waltham, MA, USA) in the wavelength range of 200–700 nm.

#### 2.3.4. Dynamic Light Scattering (DLS)

The hydrodynamic radii (Rh) of pure PVT and PVT–Te^0^NPs were determined by dynamic light scattering on a Photocor Compact-Z correlation spectrometer (Moscow, Russia) equipped by a 20 mV thermostabilized semiconductor laser (λ = 638 нм) at an angle of 90°. The autocorrelation function was analyzed by Dynals v.2 software (Tirat Carmel, Israel). The solutions for analysis were prepared by dissolving a 5 mg sample in 20 mL of distilled water at room temperature for 5 hours, pre-filtered through a 200 µm syringe filter. The time for each measurement was at least 200 s. The measurements were taken in triplicate and the mean was used.

#### 2.3.5. Gel Permeation Chromatography

The molecular weight distribution of the PVT was measured using a gel permeation chromatograph Shimadzu LC-20 Prominence (Kyoto, Japan) with a differential refractive index detector, Shimadzu RID-20A. The chromatographic column was Agilent PolyPore 7.5 × 300 mm, PL1113-6500 (Santa Clara, CA, USA) with an appropriate pre-column. High purity N,N-dimethylformamide was used as a mobile phase (1 ml/min). The calibration was carried out using a series of polystyrene standards, consisting of samples with molecular weights from 162 to 6,570,000 g/mol.

#### 2.3.6. Nuclear Magnetic Resonance

^1^H and ^13^C NMR spectra were recorded on a Bruker DPX-400 spectrometer (Billerica, MA, USA) at room temperature; ^1^H, 400.13 MHz; ^13^C, 100.62 MHz. The chemical shifts are given relative to the TMS.

#### 2.3.7. High Resolution–Transmission Electron Microscopy (HR-TEM)

The transmission electron microphotographs were obtained with an FEI Tecnai G2 20F S-TWIN transmission electron microscope (Hillsboro, OR, USA) according to the procedure detailed in [29]. The nanoparticle size distribution was determined by the statistical treatment of the microphotographs using Gatan DigitalMicrograph v.3.5 software (Pleasanton, CA, USA) and Microsoft Office Excel (Redmond, WA, USA). The electronograms from the transmission electron microscope were processed and indicated with Process Diffraction v.8.7.1 (Budapest, Hungary) and CrysTBox v.1.1 software (Prague, Czech Republic) and the crystallographic database JCPDS-ICDD PDF-2.

#### 2.3.8. Direct Current Electrical Conductivity Measurement

The DC conductivity was measured by a standard E6-13A teraohmmeter (Moscow, Russia) using a two-probe method within temperature range of 25–80 °C. A thermostat was used to maintain the temperature in the measuring cell. The powder samples were pressed in the form of pellets with a height of 0.2–0.6 mm and a radius of 1.5 mm. The DC conductivity was calculated using the following equation:

(1) $$\sigma =\frac{d}{RA}$$

where *A* is the cross-section area (cm^2^), *d* is thickness of the pellet (cm), and *R* is the resistance of the sample (Ω).

## 3. Results and Discussion

### 3.1. Synthesis of PVT–Te^0^NPs

**Figure 3:**
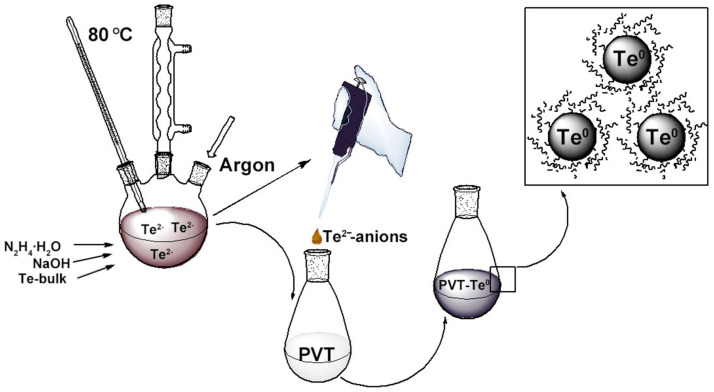
Scheme of PVT–Te^0^NPs synthesis.

Water-soluble aggregation-stable PVT–Te^0^NPs (1.4–4.3 wt% Te) were obtained by the oxidation of Te^2−^ anions to zero-valent tellurium (Te^0^) in an aqueous PVT solution. The Te^2−^ anions were pre-generated by the reduction (activation) in commercial powdered tellurium with hydrazine hydrate in an alkaline medium (Figure 3), according to the method previously proposed in [19,20].

The process resulted in the powdered tellurium being completely dissolved to form highly reactive Te^2−^ anions in accordance with Equation (2):

2Te + 4NaOH + N_2_H_4_ · H_2_O = 2Na_2_Te + N_2_↑ + 5H_2_O, (2)

The weight content of tellurium in the nanocomposites was varied by changing the PVT:Te^2−^ ratio from 1:59 to 1:17. The conversion of Te^2−^ anions to the zero-valent state varied between 84% and 80%, decreasing with increasing PVT: Te^2−^ ratio. The Te^2−^ anions obtained have a limited time stability and high sensitivity to the conditions of synthesis because of their extremely high reactivity. Presumably, the formed sodium telluride is hydrolyzed in an aqueous medium to form H_2_Te, which is further either removed from the reaction medium or oxidized by the oxygen present in the aqueous solution to Te^0^ followed by the condensation of its atoms into nanoparticles and their subsequent stabilization. Probably the reason for the decrease in the Te^2−^ anion conversion to Te^0^ is the part of H_2_Te that is hydrolyzed and then volatilized from the reaction medium. The process can be identified by the presence of a radish smell associated with H_2_Te. Under these conditions, we obtained Te^0^-containing PVT-based nanocomposites with yields of 58–71%, which are water soluble powders of grey color.

### 3.2. XRD Analysis

**Figure 4:**
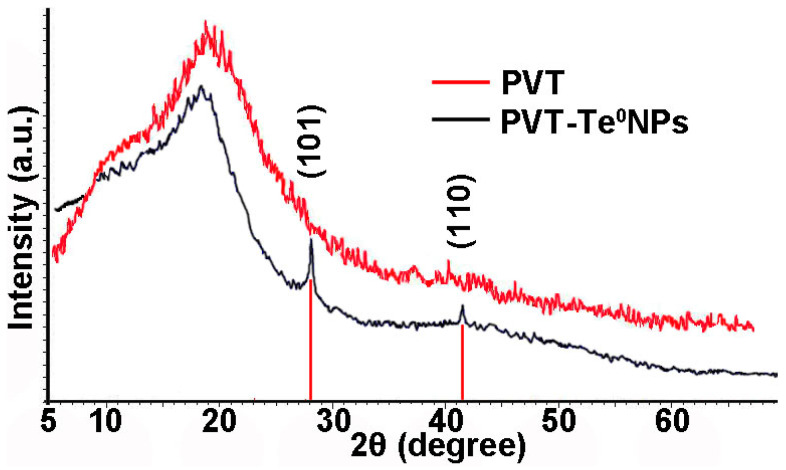
Typical XRD patterns for pure PVT and PVT–Te^0^NPs (4.3 wt% Te).

According to X-ray diffraction analysis, the PVT–Te^0^NP (1.4 wt% Te) is X-ray amorphous, whereas the nanocomposites with a higher weight content have a two-phase amorphous-crystalline structure. Their diffractograms are characterized by an amorphous halo of the PVT phase as well as a number of reflexes corresponding to the crystalline phase of Te^0^ nanoparticles (Figure 4). We assume that the formation of Te^0^NPs in the hexagonal modification under the selected soft experimental conditions is probably due to the presence of the stabilizing matrix PVT in the reaction medium. In this case, the PVT molecules are capable of limiting the growth tendency of the material, which leads to the formation of hexagonal Te^0^ nanoparticles. Moreover, even in the case of the formation of a mixture of amorphous and crystalline tellurium under low-temperature conditions, the amorphous tellurium particles, due to their extremely low stability, are prone to re-dissolution during Ostwald ripening, and the released tellurium atoms can go to the completion of the crystal lattice of more stable hexagonal tellurium particles [30,31,32].

Thus, two broadened reflexes observed at angles of 27.55° and 40.45° in diffractograms for PVT–Te^0^NPs (4.3% Te) can be ascribed to the (101) and (110) planes of the Te^0^ hexagonal lattice, respectively. The Te^0^ nanocrystallite average size in the nanocomposite calculated by the Scherer formula is 40.7 nm.

### 3.3. HR-TEM Analysis

**Figure 5:**
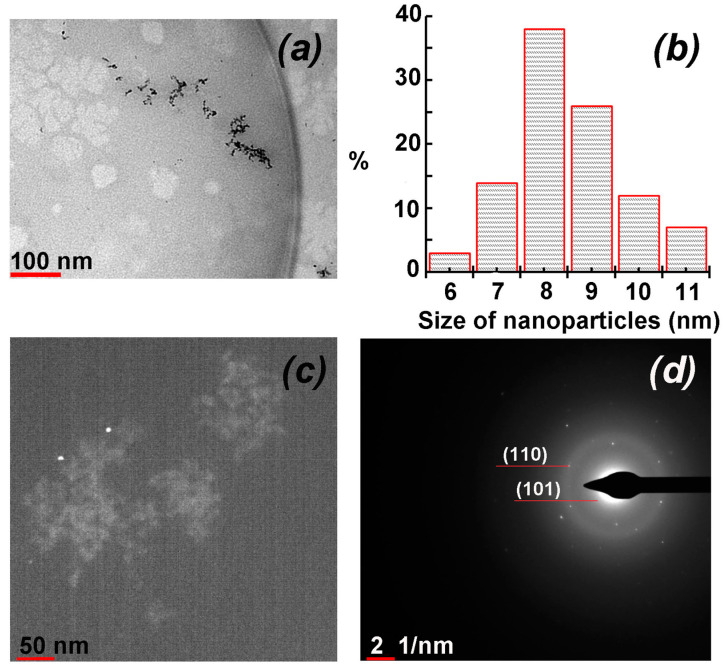
TEM image of PVT–Te^0^ nanocomposite (2.8 wt%) (**a**) in light field mode; (**b**) nanoparticle size distribution histogram of PVT–Te^0^ nanocomposite (2.8 wt%); (**c**) TEM image of PVT–Te^0^ nanocomposite (2.8 wt%) in dark field mode; (**d**) electronographic image of Te nanoparticles in PVT–Te^0^ nanocomposite (2.8 wt%).

On the basis of HR-TEM data, it was found that PVT–Te^0^NPs are formed as high-contrast particles distributed in the PVT polymer matrix with a shape close to spherical. The nanoparticles have a pronounced tendency to partially agglomerate and aggregate into chains and dimers (Figure 5a). The size of the Te^0^ nanoparticles formed in the PVT–Te^0^NPs (2.8 wt%) varies in the rather narrow range of 6–11 nm with a predominance (64%) of 8–9 nm particles and Te^0^ nanoparticle average size of 8.5 nm (Figure 5b). The dark-field images reveal that the nanoparticles are clearly visualized as they contrast significantly with the surrounding matrix, confirming their crystalline structure (Figure 5c).

The internal microstructure of the nanoparticles was also investigated by HR-TEM. The selected area electron diffraction (SAED) pattern of PVT–Te^0^NPs exhibits clear and discrete over-emission points, indicating a pronounced crystallinity of the nanoparticles (Figure 5d). The symmetrical rings with randomly distributed contrasting dots with no preferred orientation are observed in the nanocomposite (2.8 wt%) SAED pattern, indicating its polycrystalline nature. The two rings clearly visible in the SAED pattern were used as an initial data for the calculation of the interplanar distances of the nanoparticles. The distances were was found to be 3.2 Å and 2.2 Å for the first and second ring, respectively. These values are very close to the interplanar distance values of the elemental tellurium hexagonal lattice and correspond to its (101) and (110) crystallographic planes.

### 3.4. Dynamic Light Scattering Analysis

**Figure 6:**
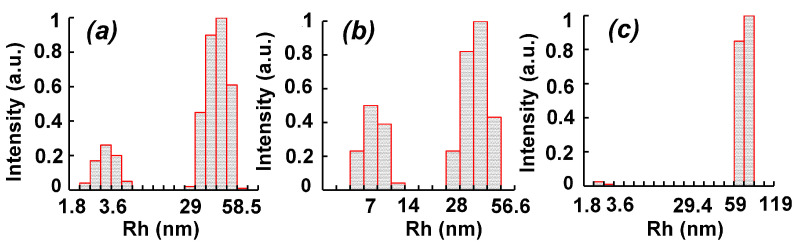
Distribution of the hydrodynamic radii (Rh) over the scattering intensity in PVT–Te^0^NP nanocomposites with a Te content of (**a**) 1.4 wt%, (**b**) 2.8%, and (**c**) 4.3%.

The study of aqueous solutions of PVT–Te^0^NPs by the DLS method shows that the particle size distribution in terms of the scattering intensity is featured by bimodality. The colloids are characterized by two particle fractions (Figure 6).

Thus, the particle fractions with a hydrodynamic radius (Rh) of 3.6 nm and 45 nm were detected in an aqueous solution of PVT–Te^0^NPs containing 1.4 wt% of Te (Figure 6a). Presumably, the first fast particle fraction is related to the presence of the individual PVT macromolecules in the nanocomposite water solution (Rh equals to 4.1 nm for PVT, whereas one is 3.1 nm for PVT–Te^0^NPs). The second particle fraction with an average Rh of 45 nm probably originates from Te^0^ nanoparticles formed in the PVT matrix or their agglomerates, which we detected by SEM. The increase in the Te weight content in the nanocomposite up to 4.3% is accompanied by a decrease in the fraction of the fast particle in its aqueous solution as well as an increase in the slow particle fraction. At the same time, there is an increase in the average Rh value of the slow particle fraction up to 75 nm, probably due to a growth of the average size of Te^0^ nanoparticles themselves (Figure 6c). In addition, a significant narrowing of the particle dispersion of both slow and fast fractions of the PVT–Te^0^NP solution with the highest Te content, as compared to the samples containing 1.4 and 2.8 wt% Te, should be noted. Thus, the minimum particle Rh range is observed to be 2.3–3.0 nm and 59–75 nm for the fast and slow particle fraction of PVT–Te^0^NPs (4.3 wt%), respectively.

### 3.5. UV-Vis Spectroscopy

**Figure 7:**
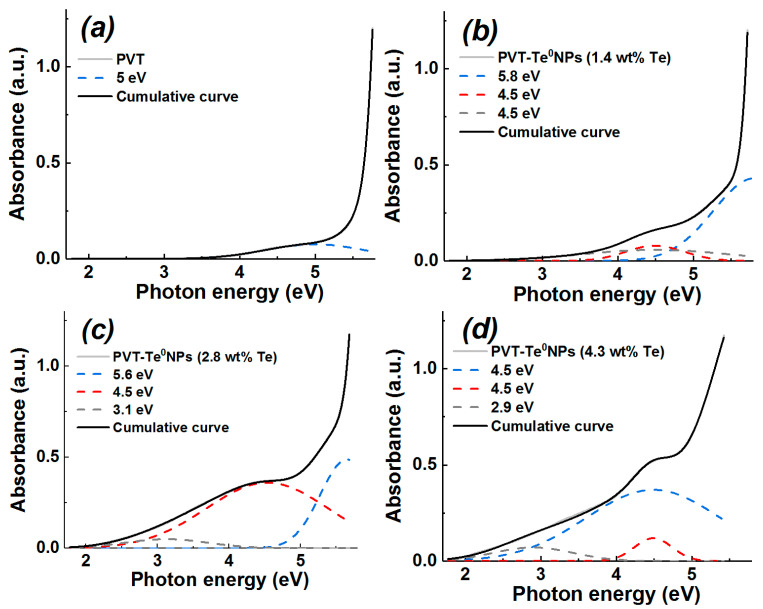
UV-Vis spectra and cumulative curves (solid lines) of 0.025% water solution of (**a**) pure PVT, and PVT–Te^0^NPs with a Te weight content of (**b**) 1.4%, (**c**) 2.8%, and (**d**) 4.3%. Photon energies corresponding to the peaks of the Gaussians are shown in the legends (dashed lines).

The study of optical absorption spectra in the range 200–700 nm of 0.025% aqueous solutions of PVT and PVT–Te^0^NPs showed that the presence of an absorption band in the region of 276 nm (4.5 eV) is typical for all the samples (Figure 7). The 5 eV absorption band in the PVT spectrum isolated by deconvolution seems to be due to the availability of the C=N chromophore group in the polymer chains, which is characterized by a band at 4.7–5.4 eV in the absorption spectrum [33]. The deconvolution also reveals the presence of three bands in the region of 3.1, 4.5, and 5.6 eV in the absorption spectra of the nanocomposites under study. In contrast to the 4.5 eV absorption band, associated with the availability of PVT in nanocomposites and invariably present in the spectra of nanocomposites with different content of inorganic nanophase, the other two absorption bands undergo a blue shift with an increasing Te weight content. Thus, with a change in the inorganic nanophase content from 1.4 to 4.3% Te, a shift of the absorption bands from 5.8 to 4.5 and from 4.5 to 2.9 eV is observed (Figure 7).

According to available data [34,35], the Te^0^ nanoparticles are characterized by the presence of an absorption band in the 270–300 nm region (4.6–4.1 eV). At the same time, the absorption spectrum of Te nanowires is featured by the availability of two bands in the region of 278 nm (4.5 eV) and 586 nm (2.1 eV) [36]. Hence, it can be concluded that the absorption bands in the region of 5.8–4.5 eV and 4.5–2.9 eV are directly related to the presence of Te^0^ nanoparticles in PVT–Te^0^NPs.

**Figure 8:**
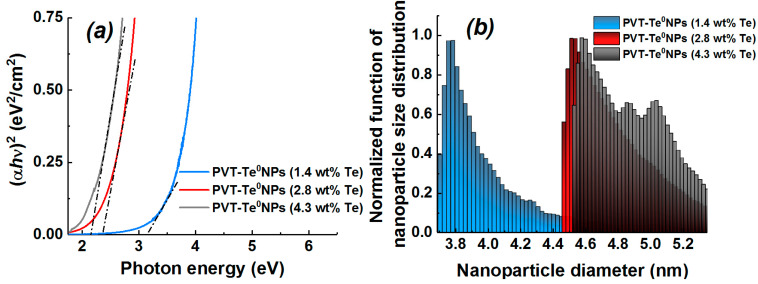
(**a**) Tauc plots for PVT–Te^0^NPs nanocomposites with different Te weight content, (**b**) dependence of the normalized nanoparticle size distribution function on nanoparticle diameter for PVT–Te^0^NPs with different Te weight content.

Based on the nanocomposite optical absorption spectra obtained, we determined their optical band gap, the average radius of nanoparticles, and the size particle distribution of the nanocomposite Te^0^ nanoparticles. The optical band gap was obtained in two ways: using the wavelength corresponding to 10% absorption in the measured optical absorption spectrum of the solutions studied [37] and by the Tauc method [38,39]. According to the Tauc method, the optical band gap of PVT–Te^0^NPs was determined by extrapolating (to the intersection with the abscissa axis) the linear sections of absorption spectra represented in Tauc coordinates (Figure 8a) using Equation (3):

αhν = A (hν − E_g_)^γ^, (3)

where α is the absorption coefficient defined by Beer–Lambert’s law, hυ is the incident photon energy, A is a constant characterizing the degree of ordering of the material structure (for calculations we assumed that A = 1), Eg is the optical band gap, γ is an index describing transition process, and γ = 1/2 for direct allowed transitions. The resulting values are shown in Table 1. As can be seen from the table, the optical band gap decreases from 3.17 to 2.16 eV (Figure 8a) or from 2.79 to 2.05 eV with increasing inorganic nanophase content in the nanocomposites studied, depending on the method of Eg determination.

As these values are larger than the band gap of bulk Te $E_{g}^{bulk}=0.335$ [40] (Figure 8a), one can assume an appearance of the quantum confinement effect resulting in an Eg increase at the transition of material from a bulk to nanoscale state (“blue shift” of Eg). The effect has been well described in the literature [41]. The possibility of setting the band gap by controlling the size of semiconductor nanoparticles has previously been reported in studies of Se colloidal solutions [42], cobalt selenide nanostructure films [43], and ZnS nanoparticles stabilized with a PVA [44]. Taking into account the approximate spherical shape of the nanoparticles, it is possible to estimate the average size of the Te^0^ nanoparticles formed according to the Equation (4) proposed by L. Brus:

(4) $$E_{g}=E_{g}^{bulk}+\frac{\hbar^{2}\pi^{2}}{2r^{2}}\left(\frac{1}{m_{e}}+\frac{1}{m_{h}}\right)-\frac{1.8e^{2}}{4\pi \varepsilon \varepsilon_{0}r}$$

where $E_{g}$ is the nanoscale Te energy band gap, $E_{g}^{bulk}=0.335\;эB$ is the energy band gap of bulk Te, ε=23 denotes the dielectric permittivity of the bulk Te, $m_{h}=m_{h}^{*}m_{0}=0.109m_{0}$ is the effective mass of the hole in Te, $m_{e}=m_{e}^{*}m_{0}=0.05m_{0}$ is the effective mass of electron in Te [40], $m_{0}$ represents the electron mass, ℏ is the reduced Planck constant, r denotes the radius of the nanoparticle, $\varepsilon_{0}$ is the electric constant, and e represents the electron charge. The values of the nanoparticle average size (diameter) calculated without considering the third term in Equation (2) are shown in Table 1. The calculation results show that the average Te^0^ nanoparticle size in the nanocomposite increases with a growing inorganic nanophase content. In this case, the value of the optical band gap, and therefore the “blue shift” is reduced. A similar trend was observed by Singh et al. in their study of the optical properties of Se quantum dots [42].

In order to determine the characteristic diameters of Te nanoparticles in the nanocomposites, the corresponding particle size distribution function curves were plotted according to the method proposed by Pesika [45]. This method makes it possible to determine the particle size distribution using the relation between the nanoparticle radius and the shift of the band gap formulated by Brus (Equation (4)):

(5) $$N\left(r\right)=-\frac{1}{V}\left[\frac{dD}{dr}\right]=-\frac{1}{\frac{4\pi r^{3}}{3}}{\left.\left[\frac{dD}{d\lambda }\frac{d\lambda }{dr}\right]\right|}_{\lambda =\frac{hc}{E_{g}(r)}}$$

where N(r) is the particle size distribution, D denotes the optical density obtained from the optical absorption spectra, r is the nanoparticle radius, V is the volume of a spherical nanoparticle, λ is a wavelength, $E_{g}$ denotes the optical band gap of the nanoscale semiconductor, h is a Plank constant, and c is a light constant.

Figure 8b shows the calculated curves of the particle size distribution function of PVT–Te^0^NPs as histograms. It was found that the PVT–Te^0^NPs with inorganic nanophase content of 1.4 and 2.8% are characterized by log-normal size distributions with maximum values corresponding to 3.8 and 4.5 nm, respectively. The particle size distribution for the PVT–Te^0^NP (4.3 wt%) has a more complex multimodal structure. The highest value of the distribution function is observed at 4.6 nm, while the peaks of the other two modes correspond to 4.9 and 5.0 nm. The resulting Te^0^ nanoparticle diameters are of the same order of magnitude as their dimensional characteristics determined using HR-TEM and the Brus equation (Equation (4)).

### 3.6. DC Electrical Conductivity

**Figure 9:**
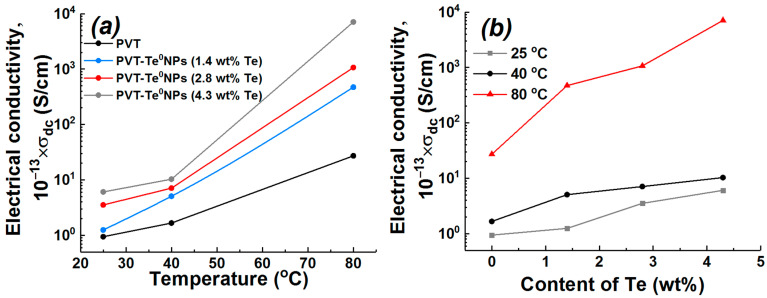
The dependence of electrical conductivity (**a**) on temperature and (**b**) on Te weight content for PVT and PVT–Te^0^NPs.

The dependences of the specific DC electrical conductivity of PVT and PVT–Te^0^NPs on temperature (in the temperature range of 25–80 °C) and the weight content of the inorganic nanophase were experimentally obtained to determine the presence of a particle dimensional effect on the electrical conductivity of the nanocomposites under study (Figure 9).

The electrical conductivity values and the nature of its change with increasing temperature allow the initial PVT polymer to be classified as a dielectric (Figure 9a). The introduction of Te nanoparticles into the PVT matrix was found to result in an increase in the DC electrical conductivity of the nanocomposite. The electrical conductivity of the composite increases as the weight content of the inorganic nanophase rises (Figure 9b). At the same time, the electrical conductivity of the nanocomposites grows with increasing temperature (Figure 9a). It should be noted that the electrical conductivity of PVT–Te^0^NPs with a Te content of 2.8 and 4.3% becomes greater than 10^−10^ S/cm at 80 °C, i.e., it overcomes the conventional dielectric-semiconductor boundary noted in the classification of materials by electrical conductivity value [46]. The behavior of the samples DC conductivity is common to dielectrics and semiconductors. The exponential growth of conductivity with an increasing temperature is characteristic of both semiconductor nanoparticles [47,48] and nanocomposites of dielectric polymers and semiconductor nanoparticles [49,50]. The variation of DC conductivity of PVA–Se nanocomposite with temperature showed the presence of nearly linear part and the part well described by the Vogel–Fulcher–Tammann relationship [49]. Sinha et al. attributed the conductivity behavior to the thermally activated hopping transport ions decoupled from the matrix and 3D variable range hopping of charge carriers. According to the literature data, the authors of paper [50] observed conductivity behavior most similar to the one we obtained. The introduction of TiO_2_ nanoparticles into PVA–PEG–PVP matrix resulted in a grow of DC electrical conductivity. Meanwhile, the increase in weight content of inorganic nanophase from 2% to 8% also led to a rise in the nanocomposite DC conductivity. The authors have explained the behavior of PVA–PEG–PVP/TiO_2_ nanocomposite conductivity by a reduction in their band gap and therefore thermally activated enhancement of charge carriers hopping motion between trapped sites.

The PVT–Te^0^NPs nanocomposite is semiconductor nanoparticles non-uniformly distributed in the volume of the dielectric matrix. It can be represented as an organic–inorganic system of components, each exhibiting different physical properties (in particular, electrical conductivity) due to its microscopic characteristics. The macroscopic physical properties of a nanocomposite are due to its microstructural features [51]. Not only the matrix and nanofillers, but also the interphase, which is a system of formed interfaces at the nanoparticle-matrix interfaces, are considered to be components of a nanocomposite. Therefore, an increase in temperature does not uniformly affect the electrical conductivity of the nanocomposite components due to the different nature of the nanocomposite components. In a dielectric, for example, as the temperature rises, some of the electrons gain energy and participate in electrical conductivity. This happens more efficiently in a semiconductor. The transport of charge carriers in the interphase is enabled by an electron tunneling effect. This effect is particularly influential when the distance between the nanoparticles is significantly reduced. However, at high temperatures, the interface conductivity decreases due to an increase in the number of electron collisions. In addition, as the temperature rises, the thickness of the interface layer may decrease, resulting in a reduction in electrical conductivity.

In the case of PVT–Te^0^NPs, the increase in their electrical conductivity, in comparison with the matrix, can be explained by the appearance of the charge-carrier transport across the crystalline semiconductor and interface conduction due to the electron tunneling effect. As the weight content of the inorganic nanophase increases, the nanoparticles become larger, i.e., the regions of successful charge carrier transport through the semiconductor increase with a simultaneous decrease in the interphase effect. This leads to an increase in the degree of ordering in the composite system, which translates into the increased electrical conductivity. In addition, the observed increase in DC electrical conductivity may be related to the possible formation and increase in the number of local conductive channels created when the average interparticle distance decreases [52].

## 4. Conclusions

Thus, we have first obtained the nanocomposites of an original poly(1-vinyl-1,2,4-triazole) polymer matrix and Te^0^ thermoelectric nanoparticles with varying amounts of inorganic phase in the range of 1.4–4.3%. The synthesized nanocomposites have been characterized using a set of complementary modern methods (HR-TEM, XRD, DLS, UV-Vis spectroscopy). Nanocomposites have been found to form as nanoparticles dispersed in a polymeric matrix and sized between 4 and 18 nm. Using the optical spectroscopy data as well as computational values calculated by the Brus formula and the Pesika method, the mean diameters and characteristic nanoparticle diameters of the nanoparticle fractions present in the composite, in the range of 4.2–5.1 and 3.8–5.0 nm, were determined for the nanocomposites containing 1.4%, 2.8%, and 4.3%. Based on the temperature dependence of the DC electrical conductivity in the range 25–80 °C, it was found that the introduction of the inorganic nanophase in the PVT dielectric polymer as well as an increase in the average size of Te^0^ nanoparticles leads to an increase in electrical conductivity over the entire temperature range. Herewith, the DC electrical conductivity values of PVT–Te^0^NPs with a Te weight content of 2.8% and 4.3% become higher than 10^−10^ S/cm at 80 °C, i.e., they exceed the conventional boundary of dielectric–semiconductor in the classification of materials by electrical conductivity value. The results obtained suggest that varying the Te weight content in the synthesis process of PVT-based nanocomposites enables one to obtain nanocomposites with a required value of electrical conductivity. Taking into account the rather high Seebeck coefficient of Te^0^ nanoparticles and probably the low thermal conductivity provided by dielectric polymer PVT matrix, this could result in the directional design of thermoelectric nanocomposites with a high thermoelectric figure of merit.

## Figures and Tables

**Table 1:** Calculated values of the optical band gap, the mean nanoparticle diameter, and the “blue shift” for PVT–Te^0^ nanocomposites. Note: $E_{g}^{T}$: optical band gap calculated by Tauc method (eV); $D^{BT}$: mean nanoparticle diameter determined by the Brus equation using the value $E_{g}^{T}$ (nm); ${BS}^{T}$: “blue” shift of $E_{g}^{T}$ value from optical band gap of bulk Te (eV); $E_{g}^{10}$: the optical band gap calculated by the Tauc method (eV); $D^{B10}$: mean nanoparticle diameter determined by the Brus equation using the value $E_{g}^{T}$ (nm); ${BS}^{10}$: “blue” shift of $E_{g}^{T}$ value from the optical band gap of bulk Te (eV).

Nanocomposite	Tauc Method			10% Absorbance Method		
	$E_{g}^{T}$	$D^{BT}$	${BS}^{T}$	$E_{g}^{10}$	$D^{B10}$	${BS}^{10}$
PVT–Te^0^ (1.4 wt% Te)	3.17	2.9	2.84	2.79	4.2	2.46
PVT–Te^0^ (2.8 wt% Te)	2.36	3.0	2.03	2.11	5.0	1.78
PVT–Te^0^ (4.3 wt% Te)	2.16	3.2	1.83	2.05	5.1	1.72

## Data Availability

The data presented in this study are available from the corresponding author upon request.
